# The influencing factors of hearing protection device usage among noise-exposed workers in Guangdong Province: a structural equation modeling-based survey

**DOI:** 10.1186/s12889-024-18428-7

**Published:** 2024-04-15

**Authors:** Jianyu Guo, Linyan Shu, Wei Wen, Guoyong Xu, Lichun Zhan, Maosheng Yan, Taihua Long, Zhixing Fan, Junle Wu, Bin Xiao

**Affiliations:** 1 Guangdong Province Hospital for Occupational Disease Prevention and Treatment, Guangzhou, China; 2 Guangdong Medical University, Dongguan, , China; 3 Guangdong Pharmaceutical University, Guangzhou, China

**Keywords:** Knowledge, Attitude, Comfort, HPD, Hearing conservation, Structural equation model, Influencing factors, Behavior

## Abstract

**Background:**

There are numerous complex barriers and facilitators to continuously wearing hearing protection devices (HPDs) for noise-exposed workers. Therefore, the present study aimed to investigate the relationship between HPD wearing behavior and hearing protection knowledge and attitude, HPD wearing comfort, and work-related factors.

**Method:**

A cross-sectional study was conducted with 524 noise-exposed workers in manufacturing enterprises in Guangdong Province, China. Data were collected on hearing protection knowledge and attitudes, HPD wearing comfort and behavior, and work-related factors through a questionnaire. Using structural equation modeling (SEM), we tested the association among the study variables.

**Results:**

Among the total workers, 69.47% wore HPD continuously, and the attitudes of hearing protection (26.17 ± 2.958) and total HPD wearing comfort (60.13 ± 8.924) were satisfactory, while hearing protection knowledge (3.54 ± 1.552) was not enough. SEM revealed that hearing protection knowledge had direct effects on attitudes (β = 0.333, *p* < 0.01) and HPD wearing behavior (β = 0.239, *p* < 0.01), and the direct effect of total HPD wearing comfort on behavior was β = 0.157 (*p* < 0.01). The direct effect also existed between work shifts and behavior (β=-0.107, *p* < 0.05). Indirect relationships mainly existed between other work-related factors, hearing protection attitudes, and HPD wearing behavior through knowledge. Meanwhile, work operation had a direct and negative effect on attitudes (β=-0.146, *p* < 0.05), and it can also indirectly and positively affect attitudes through knowledge (β = 0.08, *p* < 0.05).

**Conclusion:**

The behavior of wearing HPD was influenced by hearing protection knowledge, comfort in wearing HPD, and work-related factors. The results showed that to improve the compliance of noise-exposed workers wearing HPD continuously when exposed to noise, the HPD wearing comfort and work-related factors must be taken into consideration. In addition, we evaluated HPD wearing comfort in physical and functional dimensions, and this study initially verified the availability of the questionnaire scale of HPD wearing comfort.

**Supplementary Information:**

The online version contains supplementary material available at 10.1186/s12889-024-18428-7.

## Background

Industrial noise is the most common occupational disease hazard that can adversely affect the health of workers, of which occupational noise-induced hearing loss (ONIHL) is the most common occupational disease in the world [1]. The World Health Organization estimates that the number of people with disabling hearing loss (DHL) at global and regional levels will exceed 900 million in 2050 [2]. In China, occupational noise-induced hearing loss ranks first in the number of reported cases of key occupational diseases in Guangdong Province, and it is also the top priority of occupational disease prevention and control in Guangdong province [3, 4].

NIOSH recommends removing hazardous noise from the workplace whenever possible, implementing administrative controls, and using hearing protectors in those situations where dangerous noise exposures have not yet been controlled or eliminated [5]. Although noise control at the source is the most effective way to control occupational noise, it is difficult for some companies to fundamentally control noise due to limitations such as production processes and equipment costs [6]. ISO-1999 [7] and country/region-specific regulations [8] recommend considerations ranging from the definition of acceptable noise limits to hearing conservation programs, including the use of HPD. Hearing protection devices such as earplugs or earmuffs are widely used to reduce noise exposure, and it has been shown [9] that the standardized use of personal hearing protection devices is beneficial in reducing the risk of noise-induced hearing loss. Furthermore, previous research [10] indicated that an increase in the use of HPD at work in over-exposure to hazardous noise areas showed a decrease in hearing loss. Therefore, exploring the influencing factors of whether continuous wearing HPD in over-exposure to hazardous noise workplace plays a particularly important role in achieving the protection of workers from noise hazards.

The latest review [11] showed that the five main factors affecting HPD wearing by workers are sociodemographics, context, interpersonal relationships, cognitive perceptions, and health-promoting behaviors. Taban [12] and Meira [13] et al. pointed out that workers’ age, education, gender, economic level, etc., were significantly related to the use of HPDs. Beyond this, the understanding of the relationship between risk cognition, knowledge, and protective behaviors in the noise environment also plays an important role in occupational risk control and management, and risk perception is also related to workers’ experiences, safety behaviors, and values and beliefs [14, 15]. Among these previous studies, we found that demographic characteristics, hearing protection knowledge, and attitudes are the influencing factors of HPD-wearing behavior. However, due to differences such as the diverse industries and jobs in the study population, there are still conflicting results regarding the effect of individual characteristics on workers’ use of HPDs. According to the survey of numerous noise-exposed workers in different factories, we noticed that workers of non-fixed site operation or rotating shift could not keep wearing HPD when working in over-exposure to hazardous noise environments. In addition, hearing protection training [16], peer support [17], social norms [18], and business management [19] are also important factors affecting the use of HPD by workers. These factors are all associated with work. Therefore, we assume that work-related factors, such as work operation (that is work with fixed site operation and non-fixed site operation), work shifts (that is a system of working hours in which different workers take turns working at different times of the day, include night shift), colleagues’ influence and so on are potential influencing factors of HPD-wearing behavior.

In addition, another barrier to the continued use of hearing protectors is the lack of comfort in wearing them, and there is evidence that comfort is an important consideration for HPD use in noise-exposed workers [20, 21]. The “Guidelines for the Selection of Hearing Guards” (GB/T 23466 − 2009) propose that the selection principles of hearing guards include safety and health, suitability, and comfort. However, comfort is complex and multidimensional because it involves subjective feelings and emotions that are difficult to define and characterize with subjective and objective measures [22]. Often, the enterprise dose not carry out standardized training for the staff or the staff itself does not use the correct way to wear earplugs, which may lead to uncomfortable wearing of the ear protector, and hearing protection will rapidly become ineffective [23]. Studies have shown that the use of HPDs affects the effective communication of workers when using them [24]. At the same time, workers feel that using HPDs cuts them off from others and makes them feel isolated. In addition, some workers have reported that wearing HPDs for a long time can cause discomfort, such as pain and stuffiness. To consider comfort from a more comprehensive perspective, we describe comfort specifically in terms of both physical and functional dimensions in this study, according to the study of Doutres [25] et al.

Considering that there are plenty of variables included in this study, the method of structural equation modeling (SEM) is more suitable, which can compensate for the shortcomings of traditional statistical methods. Compared to the traditional multivariate statistical method, SEM can not only identify the factors but can also elucidate the complex relationship involved in the process. In addition, it can explore causal relationships between latent variables and quantitatively assess the direct and indirect effects of variables [26–28]. It can also analyze correlations among structures consisting of multiple variables simultaneously and clearly show the strength of each correlation [29]. The SEM method has been widely used in psychology, behavioral and social sciences, and other fields [30, 31].

Overall, previous studies of HPD wearing behavior focused on demographics, internal relationships, cognitive perceptions, and health-promoting behaviors but neglected the influences of work-related factors, especially work shifts and work operations. In addition, they used traditional statistical methods to explore influencing factors but ignored the interaction between different factors and the magnitude of the influencing effect. Accordingly, in this study, we included work-related factors, and SEM was used to explore the relationship among variables. Based on the above research and theoretical foundations, we hypothesized that noise-exposed workers who have good knowledge, positive attitude, and comfort feeling of wearing HPD are more likely to wear HPD continuously. We also hypothesized that workers with old age, high education, fixed-day shift, fixed site operation, and attending hearing protection training, and colleague continuous wearing would tend to wear HPD continuously. Moreover, hearing protection knowledge and attitude may have mediating effects.

Based on the effects of each factor and their magnitudes, the study was designed to propose targeted interventions and provide a scientific reference to strengthen the continuous wearing of HPD in over-exposure to hazardous noise workplaces and effectively reduce the incidence of noise-induced hearing loss.

## Methods

### Participants and data collection

Overall, 524 noise-exposed workers of three manufacturing enterprises in Guangdong Province (power plant enterprise, furniture manufacturing, and automotive manufacturing enterprises) were surveyed in this study during 2022. The median L_EX,8 h_ (normalized continuous A-weighted sound pressure level equivalent to an 8 h working-day) noise exposure level of the workers is 84.8(80.7 ∼ 96.7)dB(A). HPDs were provided and required to be worn in the over-exposure to hazardous noise environments in these enterprises, but participants were not penalized if they did not wear them. According to the content of work, the work shifts are divided into fixed-day shifts and rotating shifts.

The sampling size was calculated using the population proportion statistical formula [32]:

N = Z^2^P (1 − P)/d^2^ (Z = 1.96, *P* = 60.2%, d = 0.05),

where Z = critical value corresponding to 95% confidence level = 1.96; d = absolute precision (select a precision of 5% if the prevalence of the disease is going to be between 10% and 90% [33]) = 0.05; *P* = proportion with parameter (the awareness of hearing protection knowledge = 60.20%, which was from a previous study [34]). Considering a 20% nonresponse rate, the minimum required sample size was 441. A sufficient sample size ensures the credibility of the research results [35].

### Measures and data collection procedure

In this study, we prepared a questionnaire developed by researchers based on an extensive literature review ^[21–22,40−46]^. After conducting a presurvey before the formal survey, the questionnaire was revised according to its results (supplementary file 2). Questionnaires were distributed by the investigators who were all occupational health doctors with very rich experience and had undergone unified training and assessment. Before the survey began, we explained the purpose and significance of the study to the subjects, persuaded them to complete each question carefully according to the actual situation, and provided professional staff to assist them in completing the questionnaire. The overall Cronbach’s α coefficients of this questionnaire was 0.897 (> 0.70) and the Kaiser‒Meyer‒Olkin (KMO) was 0.876, with both Bartlett’s spherical test *p* < 0.05, which demonstrated a satisfactory level of reliability and validity.

The main contents include:

1. **Demographic**: The demographic questionnaire included information on age and education. There were four choices for age (< 30, 30 ∼ 39, 40 ∼ 49, > 50 years), and education was recorded as high school degree and below or above high school degree.
2. **Work-related factors**: The year of work, work operation, work shifts, whether participants had joined in hearing protection training, and colleagues around participants wearing HPDs in over-exposure to hazardous noise workplaces (colleagues influence) were included in the work-related factors questionnaire.
3. **Hearing Protection Device wearing behaviors**: HPD wearing behavior was evaluated by calculating the ratio of HPD wearing time and noise exposure time (0%∼100%), which was divided into intermittent wearing (< 90%) and continuous wearing (≥ 90%) [36, 37].
4. **Hearing protection knowledge and attitude scale** (supplementary file 1): (I) The knowledge dimension was measured by 7 items; correct answers received “1” points, and incorrect answers or unclear answers received “0” points. The total score range is 0 ∼ 7. (II) The attitude dimension was measured by 6 items, according to the Likert five-level score, from “strongly disagree” to “strongly agree” (1–5). Higher scores indicated better knowledge and attitudes. The total score range is 6 ∼ 30. The total score rate was calculated with the following formula: The total scoring rate = (Mean score / Total score) $\times$ 100%. Based on the Bloom’s cutoff points, the total knowledge and attitude scores were classified into poor/negative (< 60%), fair/neutral (60–79%), and good/positive (≥ 80%) categories.
5. **Comfort scale** (supplementary file 1): A total of 17 items with 5-point response categories measure two dimensions: (1) Physical (9 questions): “pain”, “static mechanical stress” and “irritation”, etc., the total score range is 9 ∼ 45. (2) Functional (8 questions): “insertion”, “annoyance”, “inconvenience”, “stability” (holding in position), and “invasion” (inhibition of head movement), etc. [25]. The total score range is 8 ∼ 40. Higher scores indicate a more comfortable feeling.

### Statistical analysis

With Epidata 3.1 double entry, all data were stored in Microsoft Excel 2016 and imported into IBM SPSS 26.0 to perform statistical analysis. Frequencies and percentages were used to describe the demographic and work-related factor information. The scale questionnaires were described by mean$\pm$standard deviation. Skews-kurtosis tests were used to check the normality of the variables of the scale questionnaire, and this study showed a normal distribution with an absolute value of skews < 3 and kurtosis < 8 [38].

The structural equation models were built and verified using SPSS-AMOS24.0. They were constructed to determine the relationship between demographic and work-related factors, hearing protection knowledge and attitudes, HPD wearing comfort, and HPD wearing behavior. The test significant level was α = 0.05. Then, a bias-corrected bootstrap 95% confidence interval (Cl) was used to examine the significance of direct and indirect effects. The test level was α = 0.05, and the SEM parameters were estimated using the maximum likelihood method. When the effect value β < 0, the relationship was negative; when β > 0, the relationship was positive. We used the chi-square free ratio (CMIN/DF), root mean square error of approximation (RMSEA), goodness-of-fit index (GFI), adjusted goodness-of-fit index (AGFI), incremental fix index (IFI) and comparative-fit-index (CFI) to evaluate the model’s goodness of fit.

## Results

### Participants

Of the 524 participants in this study. A total of 69.47% of them wore HPDs continuously. The majority of participants were aged 30 ∼ 39 years (*n* = 238, 45.4%) and above a high school degree (72.9%). The fixed site operations were 46.4%, and non-fixed site operations were 53.6%. In addition, 50.4% of participants were fixed-day shifts, and 49.6% were rotating shifts. Table 1 shows the demographic data and work-related factor data.

**Table 1:** General participants’ information (*n* = 524)

Variables	Categories	Number of participants	Percentage%
Age	< 30	90	17.2
	30 ∼ 39	238	45.4
	40 ∼ 49	127	24.2
	> 50	69	13.2
Year of work	< 10	250	47.7
	10 ∼ 19	208	39.7
	> 19	66	12.6
Education	High school degree and below	142	27.1
	Above high school degree	382	72.9
Work operation	Fixed site operation	243	46.4
	Non-fixed site operations	281	53.6
Work shifts	Fixed-day shift	264	50.4
	Rotating shift	260	49.6
Whether participants had joined in the hearing protection training	Yes	243	46.4
	No	281	53.6
Wearing of HPD by colleagues in over-exposure to hazardous noise workplaces	Not wearing HPD	18	3.4
	Wear intermittently	144	27.5
	Wear continuously	362	69.1
HPD wearing behavior	< 90%	160	30.53
	≥ 90%	364	69.47

### Hearing protection knowledge and attitudes

This study indicated a low level of hearing protection knowledge (50.6%) and a high level of attitudes (87.2%). The maximum score of knowledge and attitude was 7 (1.3%), and 30 (19.8%), respectively. The minimum score of knowledge and attitude was 0 (3.6%), and 12 (0.2%), respectively. Only 8.8% knew “how many decibels your earplugs can drop? (K4)”, and 16.4% knew “how many decibels of noise you are exposed to that require earplugs for hearing protection? (K3)”. The specific values are shown in Table 2.

### HPD wearing comfort

More than half of the participants (70.7%) had a comfortable feeling while wearing HPD, and the functional dimension (75.4%) was higher than the physical dimension (66.6%). Communication was the greatest barrier to comfort, and only 25.8% of participants thought it was easy to communicate when wearing HPD (Table 2).

**Table 2:** Descriptive statistics for hearing protection knowledge, attitudes, and comfort of HPDs wearing

	$\bar x \pm s$	*N* (%)		$\bar x \pm s$	*N* (%)		$\bar x \pm s$	*N* (%)		$\bar x \pm s$	*N* (%)
K1	0.40 ± 0.491	211(40.3)	A1	4.47 ± 0.658	502(95.8)	P1	3.62 ± 0.919	315(60.1)	F1	3.94 ± 0.814	407(77.7)
K2	0.64 ± 0.481	335(63.9)	A2	4.52 ± 0.641	507(96.7)	P2	3.35 ± 0.980	234(44.7)	F2	3.37 ± 0.745	337(64.4)
K3	0.16 ± 0.371	86(16.4)	A3	4.28 ± 0.738	485(92.6)	P3	3.34 ± 0.991	237(45.2)	F3	3.85 ± 0.858	354(67.5)
K4	0.09 ± 0.283	46(8.8)	A4	4.29 ± 0.728	480(91.6)	P4	3.48 ± 0.928	280(53.4)	F4	4.05 ± 0.816	378(72.1)
K5	0.90 ± 0.304	470(89.7)	A5	4.50 ± 0.595	512(97.7)	P5	3.54 ± 1.014	293(55.9)	F5	3.81 ± 0.779	359(68.5)
K6	0.59 ± 0.493	308(58.8)	A6	4.11 ± 0.872	438(83.6)	P6	3.57 ± 0.907	261(49.8)	F6	3.72 ± 0.676	330(63.0)
K7	0.76 ± 0.429	397(75.8)				P7	2.84 ± 0.957	135(25.8)	F7	3.45 ± 0.668	237(45.3)
						P8	3.18 ± 0.828	181(34.5)	F8	3.59 ± 0.902	306(58.0)
						P9	3.04 ± 0.615	379(72.3)			
K	3.54 ± 1.552	50.6%	A	26.17 ± 2.958	87.2%	*P*	29.98 ± 5.601	66.6%	F	30.15 ± 4.336	75.4%

K, Total awareness rate of hearing protection knowledge; A, Total holding rate of positive attitudes toward hearing protection; P, Total comfort feeling rate of physical; F, Total comfort score rate of functional. N, The number of people who answered each question correctly in the knowledge dimension, and the number of people who scored higher than their average score on each question in attitude, physical and functional dimensions. The % of the last line was the average score as a percentage of the total score.

### Structural equation model

**Fig. 1:**
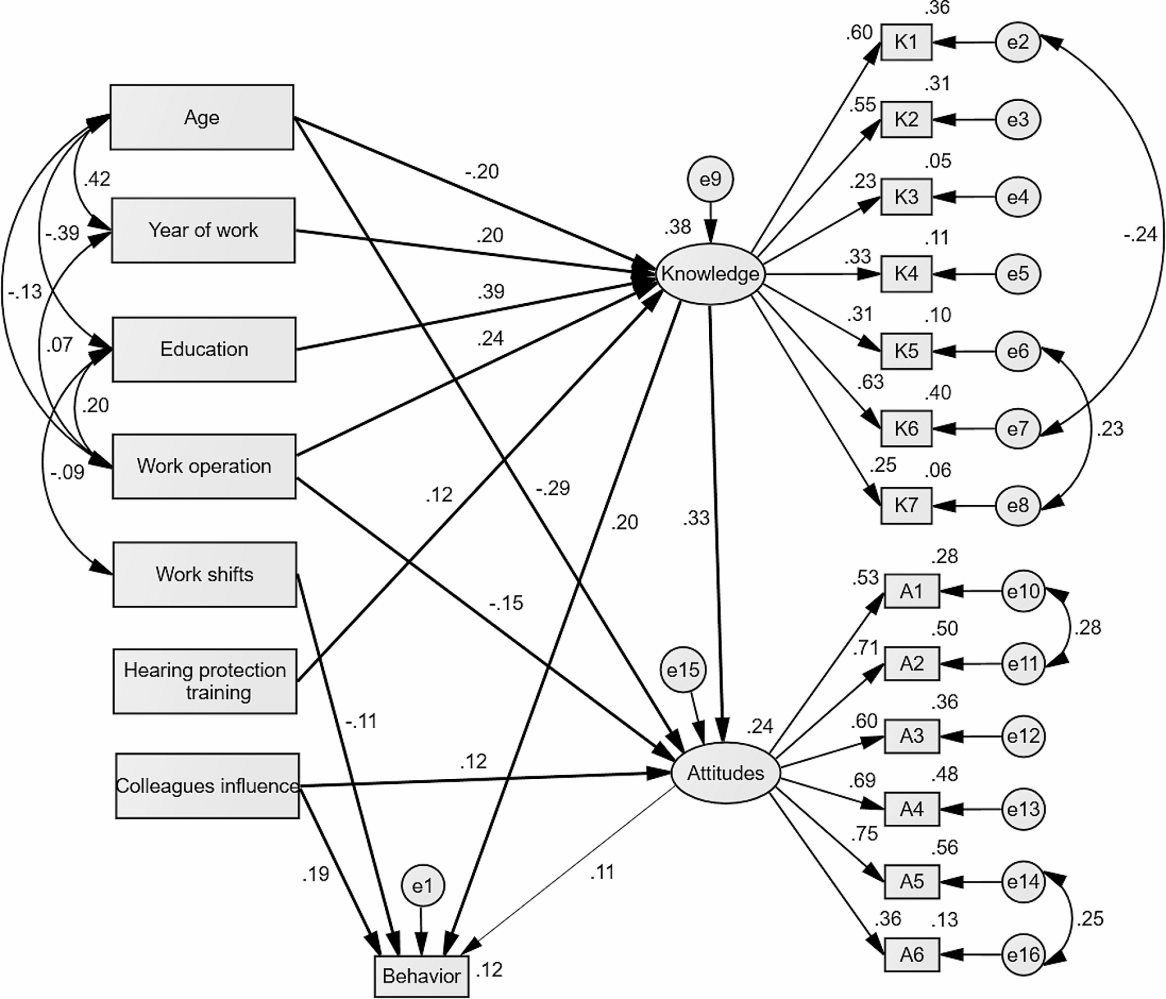
(Model 1) Testing the relationship between demographics, work-related factors, hearing protection knowledge and attitudes, and HPD wearing behavior. Rectangles indicate observed variables, ellipses represent potential variables, and circles indicate residual terms. The values of single-headed arrows represent the standardized coefficients. Fine line paths indicate non-significant coefficients

**Fig. 2:**
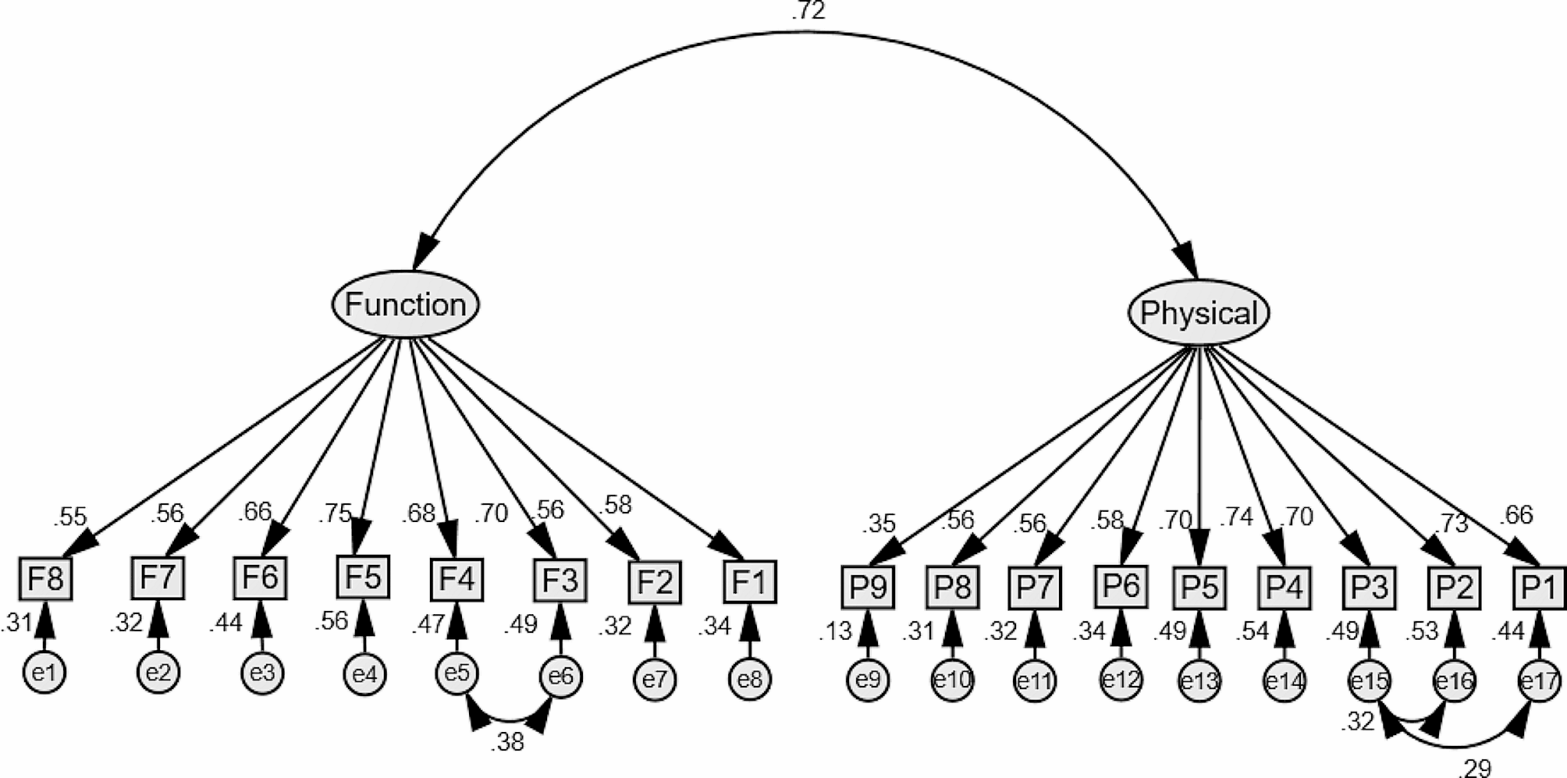
The first-order model of HPD wearing comfort and behavior (Model 2). Rectangles indicate observed variables, ellipses represent potential variables, and circles indicate residual terms. The values of single-headed arrows represent the standardized coefficients

**Fig. 3:**
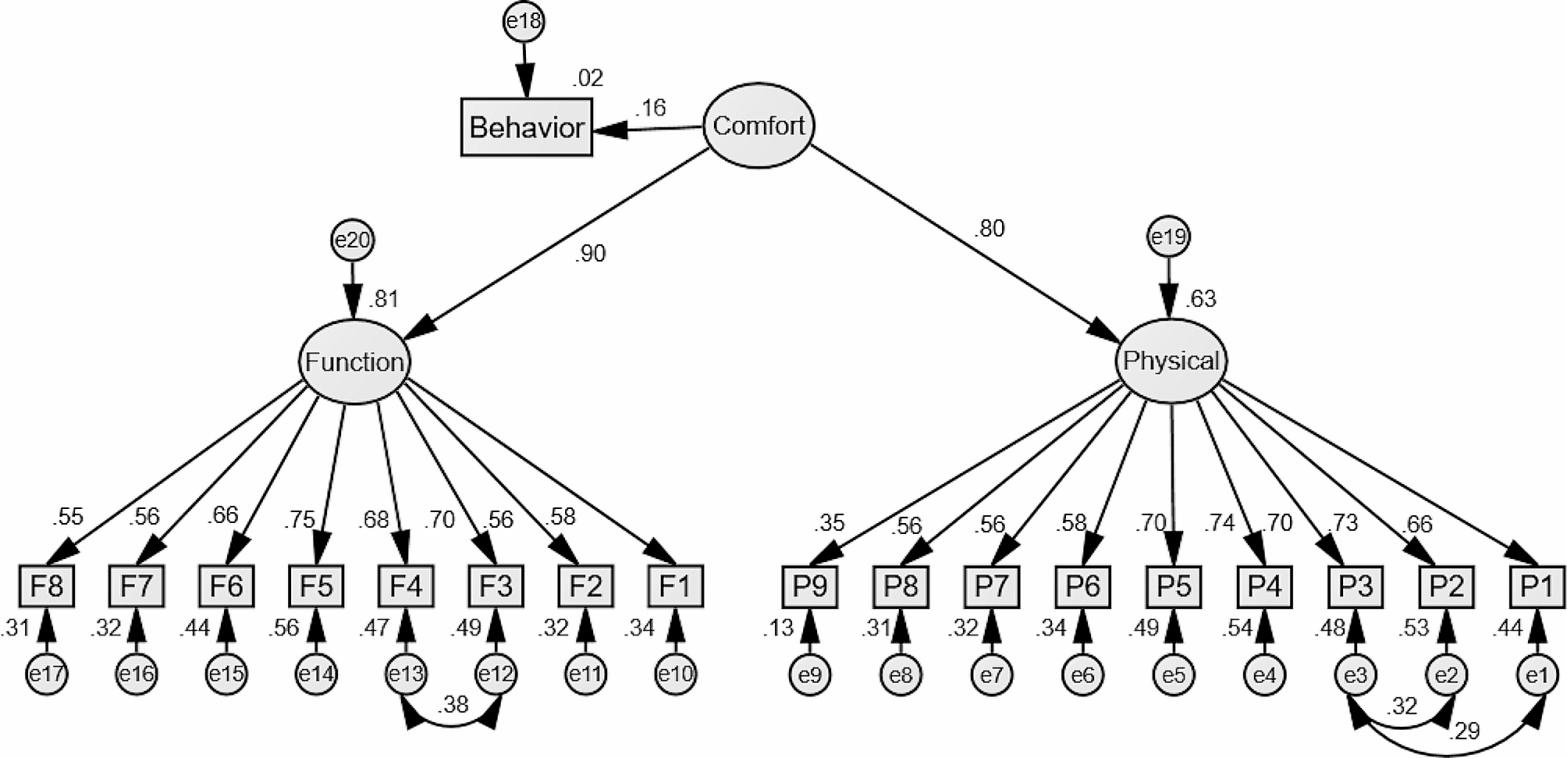
The second-order model of HPD wearing comfort and behavior (Model 3). Rectangles indicate observed variables, ellipses represent potential variables, and circles indicate residual terms. The values of single-headed arrows represent the standardized coefficients

Based on our hypotheses, there are three Models (Model 1, 2, and 3) in our study were established, where the Model 2 is the sub-model of Model 3. Model 1 (Fig. 1) shows the relationship between demographics, work-related factors, hearing protection knowledge and attitudes, and HPD wearing behavior. Model 2 is the first-order model (Fig. 2) of HPD wearing comfort which is built based on the physical and functional dimensions. And the Model 3 is the second-order model (Fig. 3) of HPD wearing comfort after adapting and modifying Model 2. Considering that there is a correlation between some residuals and observed variables, in order to make the model fit better, we adapted and modified the initial models to obtain the three models.

### Relationship between demographics, work-related factors, hearing protection knowledge and attitudes, and HPD-wearing behavior (model 1)

The model fit values in Table 3 suggest that model 1 has an ideal predictive ability or fit. As shown in Fig. 1; Table 4, (1) knowledge had a direct effect on HPD-wearing behavior and attitudes, with direct effect values of 0.201 (*p* < 0.01) and 0.333 (*p* < 0.01), respectively. (2) Age had direct and negative effects on knowledge (β=-0.198, *p* < 0.01) and attitudes (β=-0.295, *p* < 0.01), and the indirect effect on behavior was -0.081 (*p* < 0.01) through knowledge. Young workers had better knowledge, and more positive attitudes, and tended to wear HPD continuously than old workers. Likewise, education had a direct effect on knowledge with an effect value of 0.386 (*p* < 0.01), and the indirect effect was 0.092 (*p* < 0.01) through knowledge. Workers who had above high school degree were more likely to continue wearing HPD. Thus, hearing protection knowledge played a mediating role between age, education, and behavior. (3) Participants’ work shifts directly and negatively affected HPD wearing behavior (β=-0.107, *p* < 0.05). In addition, work operation had a direct and negative effect on attitudes (β=-0.146, *p* < 0.05), and it also indirectly and positively affected attitudes through knowledge (β = 0.08, *p* < 0.05). Non-fixed site operation workers had more negative attitudes, while had good knowledge.

Among them, knowledge had the greatest total effect on behavior (β = 0.239), followed by colleagues’ influence (β = 0.204). Work shifts and age had negative total effects on behavior (β=-0.107 and -0.081, respectively).

**Table 3:** The fit indices of the structural equation model (SEM)

Model	Reference index	Model 1^a^	Model 2^b^	Model 3^c^
CMIN/DF	< 5 acceptable, < 2 ideal	1.650	3.083	2.869
RMSEA	< 0.08 acceptable, < 0.05 ideal	0.035	0.063	0.060
GFI	> 0.8 acceptable, > 0.9 ideal	0.948	0.926	0.926
AGFI	> 0.8 acceptable, > 0.9 ideal	0.931	0.901	0.902
IFI	> 0.8 acceptable, > 0.9 ideal	0.814	0.930	0.929
CFI	> 0.8 acceptable, > 0.9 ideal	0.802	0.929	0.929

*P* < 0.05. a: the SEM is based on demographic, work-related factors, hearing protection knowledge and attitudes, and HPD wearing behavior. b: the first-order model of HPD wearing comfort and HPD wearing behavior. c: the second-order model of HPD wearing comfort and HPD wearing behavior.

### Relationship between HPD wearing comfort and behavior

According to model 2, the physical and functional dimensions had a significant correlation (*p* < 0.05), they belonged to the same measure model. Also, we obtained the factor loadings of each item in the physical and functional dimensions mainly between 0.55 ∼ 0.75, and only P9 was 0.35 (*p* < 0.05). The model fit values in Table 3 suggest that both model 2 and model 3 have acceptable predictive abilities or fits. The results of the analysis indicated that there was also a strong relevance between the physical and functional dimensions of HPD wearing comfort, and the factor loads were 0.80 and 0.90, respectively. HPD wearing comfort had a direct effect on behavior (β = 0.157, *p* < 0.01).

## Discussion

In the current cross-sectional study, we included the variables of “work operation, work shifts, and HPDwearing comfort”, which had rarely been studied as the influences of HPD wearing behavior before. Our study was one of the few studies that included multiple factors to analyze the interaction mechanism of HPD wearing behavior. Since HPD wearing behavior is affected by numerous factors, structural equation modeling (SEM) would be an appropriate method to achieve the study objective. Furthermore, we established a model of HPD wearing comfort in physical and functional dimensions using SEM and explored the relationship with HPD wearing behavior.

### Current status of hearing protection knowledge, attitude, and HPD wearing behavior

The findings indicated that 69.47% of noise-exposed workers consistently used HPD, which was much higher than the results of the studies conducted by Cavallari [39] (54.0%) targeting transportation road maintainers and Lv (21.6%) et al. targeting printing enterprise workers [40]. In recent years, China has taken a series of measures to prevent occupational hazards including harmful industrial noise. In Guangdong province, the government is promoting the Hearing conservation programs in workplaces. Most employers would require workers who are exposed to over-exposure to hazardous noise in workplaces to wear earplugs. Maybe this is the reason why the wearing rate of earplugs in our study is concentrated higher level (more than 90%). This study also found that it was ideal for the holding rate of positive attitudes toward hearing protection, and it was higher than the findings (76.0%) of Israel P [41]. However, our study showed that the hearing protection knowledge of participants (50.6%) was not enough, which was slightly lower than the findings (57.6%) of Gabrielle H. [42]. The results showed that the workers of the manufacturing enterprises in Guangdong, China, exhibit positive attitudes and good practices but demonstrate a relative deficiency in knowledge. The different results of the mentioned studies may be due to the safety issues in production so they tend to follow the arrangement of enterprises. Specifically, we found that nearly 90% of workers knew that exposure to industrial noise could lead to hearing loss, which may be the result of personal working experience [43]. However, we also found that less than 20% of workers were knowledgeable about the specific protective role of HPD or the appropriate circumstances for using them. The results indicated a deficiency in the hearing protection knowledge of the participants. We suspected that this was due to a lack of training in hearing protection. Meanwhile, in this study, the attitudes score of hearing protection training was the lowest, and 53.6% of participants had not participated in hearing protection training, which enhanced our assumption.

### The relationship among demographics, work-related factors, hearing protection knowledge and attitudes, and HPD wearing behavior

In our study, model 1 showed that the effect between hearing protection attitudes and HPD wearing behavior was not significant (*p* > 0.05), which may be because of the uncomfortable feeling of wearing HPD. However, hearing protection knowledge could directly and positively affect attitudes and HPD wearing behavior (*p* < 0.05). At the same time, we found that the total effect of hearing protection knowledge on behavior was the greatest (β = 0.239). Therefore, hearing protection knowledge is considered a prerequisite for continuous HPD wear.

Then, we found that the age and education of participants had indirect effects on HPD-wearing behavior through knowledge. As in the study by Taban, E [12], workers with high levels of education were more likely to wear HPDs continuously, and our study also found that the level of education could directly and positively affect hearing protection knowledge (β = 0.386, *p* < 0.001). The reason may be that higher education can motivate employees to maintain good health habits in the workplace by improving problem-solving, cognitive skills, and access to resources [44]. It is worth noting that age had direct and negative effects on hearing protection knowledge and attitudes (β=-0.198, β=-0.295, respectively, *p* < 0.001). The older the worker, the less knowledge they have about hearing protection and the worse their attitude towards hearing protection, which was in contrast to the results of Taban [12] and Marjorie [45], who showed a greater willingness to wear HPDs as workers aged. We assumed that the difference may be due to the young adult workers being inclined to follow the instructions of their employers, and they are also showing more knowledge about occupational hazards that are adverse effects of excessive noise exposure, namely, the risk of hearing loss [46, 47]. Besides, older workers may have some degree of hearing loss, and wearing earplugs can exacerbate their communication barriers. Further studies are needed to confirm this assumption.

**Table 4:** Standardized direct, indirect, and total effect in the structural equation model

variables			Standardized direct effect	Standardized indirect effect	Total effect
Age	→	Hearing protection knowledge	-0.198^**^	-	-0.198^**^
Education	→	Hearing protection knowledge	0.386^**^	-	0.386^**^
Year of work	→	Hearing protection knowledge	0.204^**^	-	0.204^**^
Work operation	→	Hearing protection knowledge	0.240^**^	-	0.240^**^
Hearing protection training	→	Hearing protection knowledge	0.115^*^	-	0.115^*^
Hearing protection knowledge	→	Hearing protection attitudes	0.333^**^	-	0.333^**^
Age	→	Hearing protection attitudes	-0.295^**^	-0.066^**^	-0.361^**^
Education	→	Hearing protection attitudes	-	0.128^**^	0.128^**^
Year of work	→	Hearing protection attitudes	-	0.068^**^	0.068^**^
Work operation	→	Hearing protection attitudes	-0.146^**^	0.080^**^	-0.066^**^
Hearing protection training	→	Hearing protection attitudes	-	0.038^*^	0.038^*^
Colleagues’ influence	→	Hearing protection attitudes	0.118^*^	-	0.118^*^
HPD wearing comfort	→	HPD wearing behavior	0.157^**^	-	0.157^**^
Hearing protection knowledge	→	HPD wearing behavior	0.201^**^	0.038^*^	0.239^**^
Hearing protection attitudes	→	HPD wearing behavior	0.113	-	0.113
Age	→	HPD wearing behavior	-	-0.081^**^	-0.081^**^
Education	→	HPD wearing behavior	-	0.092^**^	0.092^**^
Year of work	→	HPD wearing behavior	-	0.049^**^	0.049^**^
Work shifts	→	HPD wearing behavior	-0.107^*^	-	-0.107^*^
Work operation	→	HPD wearing behavior	-	0.041	0.041
Hearing protection training	→	HPD wearing behavior	-	0.027^*^	0.027^*^
Colleagues’ influence	→	HPD wearing behavior	0.191^**^	0.013^*^	0.204^**^

*p < 0.05; **p < 0.01

Another noteworthy finding of our study was the relationship between work-related factors and HPD wearing behavior. First, we found that work shifts had a direct and negative effect on HPD wearing behavior (β=-0.107, *p* < 0.05), which suggested that the rate of participants with fixed-day shifts wearing HPD continuously was higher than that of rotating shift workers. During the survey, we found that almost all managers of the enterprises work in the daytime, the less supervision or enforcement for shifts work might be the reason. For rotating shift workers, the action of wearing HPD may be forgotten frequently, and it follows that the risk of noise exposure is increased. Therefore, it is extremely important to execute HPD wearing and hearing protection training for rotating shift workers to improve their awareness of hearing protection. Second, work operations affected hearing protection knowledge positively (β = 0.204, *p* < 0.001), while it had a negative effect on attitudes (β=-0.146, *p* < 0.01). Non-fixed site operation workers with better knowledge while their attitudes were negative. We suspected that, in this study, the non-fixed site operation workers were mainly in management positions (such as team leaders, foremen, etc.), who were familiar with the process of each position and had sufficient knowledge reserves; however, due to the high activity of mobile positions and the poor compliance caused by frequent changes in the noise environment, their awareness of wearing HPD continuously in the noise environment was not optimistic. The compliance of workers with non-fixed site operations wearing HPD continuously was lower than that of fixed site operation workers. Maybe due to the implementation of the Hearing conservation programs that have enhanced the management of these enterprises, and further improved the continuous HPD wearing behavior of non-fixed site operation workers, there was no significant difference in HPD wearing behavior between the fixed and non-fixed site operations workers. Third, colleagues’ influence could directly affect HPD wearing behavior (β = 0.191, *p* < 0.001), and it also had a positive effect on behavior through hearing protection attitudes (β = 0.013, *p* < 0.05). According to the enterprises in this study, we thought it may be associated with management culture because they all had no reward and punishment rules for HPD-wearing behavior. Good health behaviors depend on a healthy supportive environment built by managers [19]. Last, both years of work and hearing protection training had indirect and positive effects on behavior (β = 0.049, β = 0.027, *p* < 0.05) through hearing protection knowledge. Hearing protection training is the main source of hearing protection knowledge obtained by workers after work, and effective training helps to promote workers’ understanding of relevant knowledge [48]. The result of years of work was consistent with the results of Feder, K [49]. As the length of working time increases, the more experienced workers are in the role, and the higher use of continuous hearing protection for inexperienced workers has a significant impact [50].

### The relationship between HPD wearing comfort and HPD wearing behavior

From the SEM results, we found that HPD wearing comfort had a direct and positive effect on behavior (β = 0.157). The higher the comfort score, the better the wearing behavior. Because the HPD protection effect is closely related to the time of HPD wear, it is better to wear HPD continuously in an over-exposure to hazardous noisy environments than to wear it intermittently [51]. However, a previous study by Samelli [52] et al. showed that the HPD attenuation value is negatively correlated with comfort; that is, the better the HPD protection effect is, the lower the comfort score. Therefore, we should pay more attention to exploring HPD wearing comfort in the future.

Furthermore, the HPD wearing comfort included two dimensions in our study: the physical and the functional dimensions [25]. The result showed that there was a strong relevance between the physical and functional dimensions of HPD wearing comfort, with factor loads of 0.80 and 0.90, respectively. Therefore, it was appropriate to establish this second order to describe the comfort of HPD wear. In our study, the results showed the physical and functional dimensions of total HPD wearing comfort were 66.6% and 75.4%, respectively. The comfortable feeling of the functional dimension was higher than that of the physical dimension, which may be due to the difference in the width and length of the ear canal in different individuals, resulting in discomfort during the use of HPDs [53]. It was necessary to focus on these specific dimensions to improve the comfort of wearing to further improve the wearing rate. In addition, we found that the feeling of isolation and communication difficulty were the greatest barriers in the physical dimension, which agreed with the view that inconvenient communication between workers is the largest obstacle to HPD wearing comfort [24]. Therefore, it was important to consider balancing comfort with communication to not over- or under-protect a worker.

### Limitations

Our study has several limitations. The main challenge with the use of SEM is the inability to infer causality in this cross-sectional study, and there is no mature reference scale to provide a more comprehensive quantitative evaluation of KAP and the comfort of HPD use. Several of the factor loadings in the present investigation were relatively low, which signaled that they had a low covariance with each other. Considering the complexity of the influencing factors of HPD use, it is necessary to increase the sample size and further explore the barriers to HPD usage. Also, the factors of the hearing status that will affect the comfort of wearing HPD were ignored. In addition, previous studies showed that the personal attenuation rating (PAR) of earplugs could influence the effect of using HPD. In the future, exploring the association between PAR and the effect of HPD would be more important. And audibility is also important to consider, especially from a safety standpoint.

## Conclusion

The results showed that hearing protection attitudes and HPD wearing behavior were satisfactory among Chinese manufacturing workers in Guangdong, while their hearing protection knowledge was not enough. In addition, work shifts had direct effects on HPD-wearing behavior, rotating shift workers with more poor behavior than fixed-day shift workers. And workers who with long working years, had joined in the hearing protection training or colleagues’ influence would be more likely to wear HPD continuously. Besides, the results further proved that HPD wearing comfort was one of the most important influences promoting the continuous use of HPD. Meanwhile, hearing protection knowledge had a mediating effect on HPD-wearing behavior. Therefore, it is essential to enhance workers’ knowledge about hearing protection. Consequently, for those who wear HPD intermittently, efforts to change the arrangement of work and improve the comfortable perceptions related to the wearing of HPD may be necessary.

### Electronic supplementary material

Below is the link to the electronic supplementary material.

Supplementary Material 1

Supplementary Material 2

Supplementary Material 3

## Data Availability

The datasets used and/or analyzed during the current study are available from the corresponding author upon reasonable request.

## Abbreviations

HPDs: Hearing Protection Devices
ONIHL: occupational noise-induced hearing loss
DHL: disabling hearing loss
SEM: Structural Equation Modeling
KMO: Kaiser‒Meyer‒Olkin
CMIN/DF: Chi-square Free Ratio
RMSEA: Root mean square error of approximation
GFI: Goodness-of-fit Index
AGFI: Adjusted goodness-of-fit index
IFI: incremental fix index
CFI: Comparative fit index
PAR: personal attenuation rating
