# Supplementary material for: The influencing factors of hearing protection device usage among noise-exposed workers in Guangdong Province: a structural equation modeling-based survey
Source: BMC Public Health. 2024 Apr 15;24:1044. doi: 10.1186/s12889-024-18428-7 (PMC11017596; doi:10.1186/s12889-024-18428-7)
Supplement: Supplementary file 1 — Supplementary Material 1 [file 12889_2024_18428_MOESM1_ESM.pdf]

Supplementary table 1 Hypothesis of latent and observed variables

Questionnaire scale	Latent variables	Observed Valuables	Reference
Hearing Protection Knowledge and Attitude	Knowledge	K1. Do you know how many decibels the "noise-exposed work" is greater than or equal to?<br>K2. Do you know the noise range of the over-exposure to hazardous noise workplaces you frequent?<br>K3. Do you know how many decibels of noise you are exposed to that require earplugs for hearing protection?<br>K4. Do you know how many decibels your earplugs can drop?<br>K5. Can industrial noise cause hearing loss or noise-induced hearing loss?<br>K6. Can noise-induced hearing loss be restored?<br>K7. Can a hearing test assess hearing loss?	(Thepaksorn et al. 2018), (Crandell et al. 2004), (Okpala, 2007)
	Attitude	A1. Working in over-exposure to hazardous noise environment can lead to hearing loss, I agree with it.<br>A2. Working in an over-exposure to hazardous noise environment, it is necessary to use earplugs.<br>A3. Even if wearing earplugs makes an impact on my communication, I still need to wear them.<br>A4. If earplugs are not mandatory, I will insist on wearing them.<br>A5. I think hearing tests are essential.<br>A6. I think hearing protection training is required to use the earplugs well.	(Saunders et al. 2014), (Nyarubeli et al. 2020), (Tantranont et al. 2009)
Comfort	Physical dimension	P1. Painless/Painful<br>P2. Ear open/Ear block<br>P3. Ear empty/Ear full<br>P4. Cold/Hot<br>P5. No itching/Itching<br>P6. No feeling isolation/Feeling isolation<br>P7. Easy to communicate/Difficulty in communication<br>P8. Breathable/Not breathable<br>P9. Tight/Loss	(Byrne et al. 2011), (Doutres et al. 2019), (Tantranont et al. 2009), (Arezes & Miguel, 2002), (Li, 2013)
	Functional dimension	F1. Freedom/Limitation<br>F2. Acceptance/Unacceptance<br>F3. Wearing easy/Wearing difficult<br>F4. Simple/Complicated	

F5. Good fit/Poor fit
F6. Convenient/Inconvenient
F7. Soft/Hard
F8. Stability/Instability
