# Supplementary material for: The influencing factors of hearing protection device usage among noise-exposed workers in Guangdong Province: a structural equation modeling-based survey
Source: BMC Public Health. 2024 Apr 15;24:1044. doi: 10.1186/s12889-024-18428-7 (PMC11017596; doi:10.1186/s12889-024-18428-7)
Supplement: Supplementary file 2 — Supplementary Material 2 [file 12889_2024_18428_MOESM2_ESM.pdf]

The PILOT study:

The PILOT study:

The Cronbach's α coefficients of the hearing protection knowledge and attitudes scale and the comfort scale were 0.763 and 0.897, respectively. Kaiser–Meyer–Olkin (KMO) is 0.801 and 0.918, respectively, with both Bartlett's spherical test $p<0.05$, with both Bartlett's spherical test $p<0.05$, suggesting good internal consistency reliability and structural validity.

The score rate of K5 is 99.23%, nearly all the workers can correctly response this question. Combined the scale after the item is deleted, the influence of the Cronbach's α coefficients in this questionnaire was only 0.02 (supplementary table 2). Therefore, we decided to deelete the K5.

The revised Cronbach's α coefficients of the hearing protection knowledge and attitudes scale and the comfort scale were 0.761 and 0.897 (>0.70), Kaiser–Meyer–Olkin (KMO) is 0.808 and 0.918, respectively, with both Bartlett's spherical test $p<0.05$, suggesting good internal consistency reliability and structural validity.

Supplementary table 2 Hypothesis of latent and observed variables

Questionnaire scale	Latent variables	Observed Valuables	The scale after the item is deleted
Hearing Protection Knowledge and Attitude	Knowledge	K1. Do you know how many decibels the "noiseexposed work" is greater than or equal to?	0.756
		K2. Do you know the noise range of the noisy areas you frequent?	0.750
		K3. Do you know how many decibels of noise you are exposed to that require earplugs for hearing protection?	0.766
		K4. Do you know how many decibels your earplugs can drop?	0.765
		K5. Do you know that industrial noise is harmful the longer the exposure time?	0.761
		K6. Can industrial noise cause hearing loss or noise hearing loss?	0.756
		K7. Can noise deafness be restored?	0.756
		K8. Can a hearing test assess hearing loss?	0.756
	Attitude	A1. Working in a noisy environment can lead to hearing loss, I agree with it.	0.737
		A2. Working in a noisy environment, it is necessary to use earplugs.	0.724
		A3. Even if wearing earplugs makes an impact on my communication, I still need to wear them.	0.738
		A4. If earplugs are not mandatory, I will insist on wearing them.	0.730
		A5. I think hearing tests are essential.	0.722
		A6. I think hearing protection training is required to use the earplugs well.	0.754
Comfort	Physical dimension	P1. Painless/Painful	0.621
		P2. Ear open/Ear block	0.66
		P3. Ear empty/Ear full	0.686
		P4. Cold/Hot	0.569
		P5. No itching/Itching	0.553

	P6. No feeling isolation/Feeling isolation	0.43
	P7. Easy to communicate/Difficulty in communication	0.405
	P8. Breathable/Not breathable	0.483
	P9. Tight/Loss	0.500
Function dimension	F1. Freedom/Limitation	0.416
	F2. Acceptance/Unacceptance	0.419
	F3. Wearing easy/Wearing difficult	0.602
	F4. Simple/Complicated	0.636
	F5. Good fit/Poor fit	0.572
	F6. Convenient/Inconvenient	0.566
	F7. Soft/Hard	0.458
	F8. Stability/Instability	0.410
