# Supplementary material for: The influencing factors of hearing protection device usage among noise-exposed workers in Guangdong Province: a structural equation modeling-based survey
Source: BMC Public Health. 2024 Apr 15;24:1044. doi: 10.1186/s12889-024-18428-7 (PMC11017596; doi:10.1186/s12889-024-18428-7)
Supplement: Supplementary file 3 — Supplementary Material 3 [file 12889_2024_18428_MOESM3_ESM.pdf]

The correlation among variables in the SEM

In supplementary table 3, there existed significant difference among hearing protection knowledge, attitudes and HPD wearing bahavior ($p<0.001$). Also, HPD wearing comfort and behavior had significant defference ($p<0.001$).

Supplementary table 3 The correlation among variables in the SEM

	Age	Education	Year of work	Work station	Work operation	Training hearing protection	HPD	Colleague influence	Knowledge	Attitude
Age	1									
Education	-0.436^{***}	1								
Year of work	0.441^{***}	0.004^{**}	1							
Work operation	-0.151^{**}	0.225^{***}	0.060	1						
Work shifts	-0.241^{***}	0.047	-0.192^{***}	0.050	1					
Training hearing protection	-0.127^{**}	0.085	-0.111^{*}	0.044	0.156^{***}	1				
HPD	-0.060	0.174^{***}	0.114^{**}	0.123^{**}	-0.080	0.110^{*}	1			
Colleague influence	-0.143^{**}	0.155^{***}	0.009	0.037	0.116^{**}	0.116^{**}	0.223^{***}	1		
Knowledge	-0.269^{***}	0.416^{***}	0.059	0.265^{***}	0.066	0.158^{***}	0.211^{***}	0.138^{**}	1	
Attitude	-0.367^{***}	0.257^{***}	-0.079	0.058	0.113^{**}	0.112^{*}	0.175^{***}	0.164^{***}	0.318^{***}	1

$\alpha = 0.05$. *$p<0.05$; **$p<0.01$; ***$p<0.001$
